# Public Key-Based Authentication and En-Route Filtering Scheme in Wireless Sensor Networks

**DOI:** 10.3390/s18113829

**Published:** 2018-11-08

**Authors:** Chuanjun Yi, Geng Yang, Hua Dai, Liang Liu, Ning Li

**Affiliations:** 1 College of Computer Science and Technology, Nanjing University of Post and Telecommunications, Nanjing 210003, China; zjyicj@126.com (C.Y.); daihua@njupt.edu.cn (H.D.); 2 Zijin College, Nanjing University of Science and Technology, Nanjing 210023, China; 3 Jiangsu Key Laboratory of Big Data Security & Intelligent Processing, Nanjing 210023, China; 4 College of Computer Science and Technology, Nanjing University of Aeronautics and Astronautics, Nanjing 210016, China; liangliu@nuaa.edu.cn; 5 Province Geomatics Center of Jiangsu, Nanjing 210013, China; tkh4@163.com

**Keywords:** wireless sensor network, security, en-route filtering, elliptic curve cryptography

## Abstract

The existing public key-based en-route filtering schemes are vulnerable to report disruption attacks or selective forwarding attacks, and they fail to consider any measure to detect and punish the malicious nodes. The authors propose a series of public key-based security mechanisms for wireless sensor networks (WSNs) in this paper, including a mechanism for verifying the partial signatures, a substitution mechanism, an effective report forwarding protocol, and a trust value-based mechanism to identify and punish the malicious nodes. Finally, the authors develop a public key-based authentication and en-route filtering scheme (PKAEF), which can resist false data injection attacks, report disruption attacks and selective forwarding attacks, and can mitigate the impact of malicious nodes. Detailed performance analysis and evaluation show that, in most cases, PKAEF outperforms previous works in terms of safety, filtering efficiency, and data availability.

## 1. Introduction

Wireless sensor networks (WSNs) have attracted a lot of attention recently, and have been applied in various applications, such as military sensing and tracking, traffic flow measuring, environmental monitoring, and real-time accident reporting etc. [1,2,3]. One major function of WSNs is to detect events of interest and deliver the event reports to the base station (BS) through multi-hop wireless paths. WSNs usually consist of numerous small sensor nodes that are resource starving and, as such, are highly constrained by battery power, storage, computation, data rate, and available bandwidth [4]. 

Due to the lack of tamper-resistant hardware, the sensor nodes in WSNs are vulnerable to be compromised. Once a node is compromised, all the security information stored in it can be used by the adversary. Then, the adversary can control the compromised node to launch various insider attacks, such as a false data injection attack [5], report disruption attack [6], selective forwarding attack [6] etc. These attacks can severely damage network function, and waste limited network resources such as energy and bandwidth, hence, severely affecting both data authenticity and availability.

To defend against the aforementioned attacks, many en-route filtering schemes have been proposed. It was a common belief that the traditional public-key cryptography (PKC) was too complex, slow and power hungry and, thus, ill-suited for use in resource-constrained WSNs [7]. Due to this, most existing en-route filtering schemes [5,8,9,10,11,12,13] are generally based on symmetric-key cryptography. However, due to the inherent limitations of symmetric-key cryptography, these schemes suffer from the lack of authentication, scalability and resilience to node compromise [14]. Therefore, some researchers [15,16] began to investigate the feasibility of using PKC on sensor platforms and showed that traditional PKC, such as RSA or elliptic-curve cryptography, is rather viable in WSNs [7]. Then, many PKC-based en-route filtering schemes [1,6,7,17,18,19,20] were proposed to filter out bogus reports at an early stage. These schemes have a few drawbacks, however; for example, some of them (the energy-aware routing and filtering scheme (ERF) [18] and the public-key based false data filtering scheme (PDF) [19]) are vulnerable to report disruption attacks and selective forwarding attacks. The digital signature assisted end-to-end data authentication scheme (DSEDA) [1] is resilient to report disruption attacks to a certain degree, but vulnerable to selective forwarding attacks. The authentication and en-route filtering scheme [6] and the location-based threshold-endorsement scheme (LTE) [7] are resilient to the two aforementioned attacks to a certain extent, however, they have high communication overhead and the security vulnerability of LTE was pointed out in [14]. Besides, none of these schemes consider detecting and punishing the malicious nodes, so the malicious nodes constantly can fool other nodes and consume the limited network resources.

The authors propose a series of public key-based security mechanisms for WSNs based on the elliptic curve Pintsov–Vanstone signature scheme (ECPVSS) [21,22] and Shamir’s secret sharing technique [23]. This study’s main contributions are summarized as follows.

First, the authors propose a mechanism for verifying the partial signatures. Accompanying this mechanism, the cluster head (CH) node can detect the invalid partial signatures at the very beginning of the report lifetime and, in this way, it can avoid generating an incorrect cluster signature for the event report.

Second, the authors design a substitution mechanism to ensure that some trusted cluster nodes will participate in report generation when the valid partial signatures are inadequate, which can ensure that enough trusted cluster nodes will participate in report generation. Additionally, with this mechanism, once a CH node refuses to send out the signed event report or sends out an event report with an illegitimate signature, a new CH node will substitute it and regenerate a signed event report.

Third, the authors design an effective report forwarding protocol that can defend against selective forwarding attacks. Unless all upstream nodes (the neighbors closer to the BS) of the sender are compromised, this scheme ensures that legitimate reports will be forwarded to the BS with less communication and computation overhead. 

Fourth, to mitigate the impact of malicious nodes, the authors propose a trust value-based mechanism to identify and punish the malicious nodes in the network. When a node has any malicious behavior, its trust value will be reduced. When the trust value of a node falls below the predefined threshold, it will be isolated.

Finally, by integrating the aforementioned mechanisms, the authors develop a public key-based authentication and en-route filtering scheme (PKAEF) to resist false data injection attacks, and report disruption attacks, and selective forwarding attacks. Detailed performance analysis and evaluation show that, in most cases, PKAEF outperforms PDF [19] and DSEDA [1] in terms of safety, filtering efficiency, and data availability.

The rest of this paper is organized as follows. The related works are reviewed in Section 2. Section 3 introduces the system model and threat models. A public key-based authentication and en-route filtering scheme is proposed in Section 4 and then, Section 5 and Section 6, analyses and evaluates of its performance in comparison to those schemes closest to that of this study’s. Finally, the conclusion of this paper is in Section 7.

## 2. Related Works

To filter out false reports as early as possible, many en-route filtering schemes have been proposed, which can be classified into two major categories: symmetric-key cryptography based schemes and public-key cryptography (PKC) based schemes. Due to the limited space, we only discuss the existing PKC-based schemes more related to this study.

To mitigate the impact of compromised nodes, Zhang et al. [7] proposed a suite of location-based compromise-tolerant security mechanisms. A notion of location-based keys (LBKs) was proposed based on a new cryptography concept called pairing, in which the private key of an individual node was bound to both its ID and geographic location. Then, an LBK-based neighborhood authentication protocol was proposed to constrain the impact of compromised nodes to their vicinity. Moreover, a location-based threshold-endorsement scheme, called LTE, was proposed to filter out bogus reports during their early transmission stages. The proposed approaches can reduce the impact of compromised nodes in the network and provide high resiliency. However, the bi-linear pairing is too expensive for low-energy sensor nodes, and localization will incur additional communication overhead. Furthermore, Duan et al. [14] pointed out that LTE cannot resist key compromise impersonation (KCI) attacks and cannot provide forward secrecy. 

Wang et al. [19] proposed a public key-based (PDF) false data filtering technique based on elliptic curve cryptography [22] and Shamir’s threshold cryptography [23]. In PDF, each sensor holds a unique share of the system secret key, and any *t* sensors can collaborate to construct the system secret key. Thus, the adversary needs to compromise at least *t* sensors to infer the system secret key. Every report is attached with a digital signature cosigned by the *t* detecting nodes using the ECPVS signature scheme [22], and any forwarding node with the system public key can verify the report. PDF can effectively reject the false data reports, but it is vulnerable to both report disruption attacks and selective forwarding attacks.

Anudeep et al. [20] proposed a hybrid approach for filtering false reports in WSNs using a hash-based short signature. In the proposed scheme, a forwarding node will detect the false report by verifying the signature of the report. Moreover, the adversary cannot forge a signature by compromising a CH node. The proposed scheme provides high security resilience, and requires slight more cost than symmetric key approaches but substantially less cost compared to public key-based approaches.

Yu et al. [6] proposed an authentication and en-route data filtering scheme for WSNs in the IoT scenario, which used the efficient ID-based signature algorithm to generate signature share and used the verifiable secret sharing technique to distribute the shares to multiple cooperative sensor nodes. When an event occurs, multiple neighboring nodes in the event area collectively generate the event report and sign the report. Finally, the signed report is forwarded to the sink through multi-path routing. The forwarding nodes verify the signature of a report with the predefined probability. The proposed scheme can defend against node compromise attacks as well as denial of service (DoS) attacks. However, multi-path routing will incur high communication overhead for forwarding a report and high computation overhead for verifying a report. The location-aware end-to-end data security scheme (LEDS) [11] saves communication overhead for forwarding a report by allowing only one node in the receiving cell to forward the report, however, each node in the receiving cell has to verify the report, which results in high computation overhead.

Geedhabhanu et al. [17] proposed an en-route data filtering scheme based on the elliptical curve digital signature algorithm (ECDSA) and the NFFS scheme, which can filter out false reports and can mitigate the impact of compromised nodes. However, ECDSA produces a 40-byte signature, resulting in large message payload. An ECPVS signature scheme [21,22] can offer smaller signature size than ECDSA. Besides, the proposed scheme is vulnerable to report disruption attacks and selective forwarding attacks.

Ferng et al. [1] proposed an enhanced and efficient cluster-based authentication protocol using digital signature rather than message authentication codes (MACs), called DSEDA. DSEDA can avoid false information at intermediate nodes, and can guarantee end-to-end data authentication with the aid of a digital signature. Besides, a report collection mechanism was proposed to filter out bogus reports as soon as possible, and a mechanism to compensate lack of legitimate secret shares was proposed to enhance data availability. However, if a CH node is compromised, it can generate false reports with legitimate signatures, which can pass the en-route filtering.

Shahzad et al. [18] presented an energy-aware routing and filtering node (ERF) selection scheme for commutative cipher-based en-route filtering (CCEF). First, the keys are disseminated to only some intermediate nodes using a probabilistic method. Then, a path from the BS to the selected CH is established with consideration of distance and energy in all sensor nodes. When a CH receives a query from the BS, it creates the final report which contains all the MACs from the participating nodes, and transmits the report to the BS. The filtering nodes, which are selected based on attack information, presence of witness key and current state of a node, verify the MACs using the property of commutative ciphers. The proposed approach can significantly prolong the network lifetime by balancing network energy consumption. However, it is vulnerable to report disruption attacks and selective forwarding attacks.

In summary, the existing PKC-based schemes have two major drawbacks: one is that they are vulnerable to report disruption attacks or selective forwarding attacks; the other is that they do not provide any measure to detect and punish the malicious nodes. To overcome these drawbacks, a public key-based authentication and en-route filtering scheme (PKAEF) is proposed in this paper, which can resist multiple types of attack such as false data injection attack, report disruption attack, and selective forwarding attack. Moreover, a trust value-based mechanism for detecting and punishing the malicious nodes is proposed, which can mitigate the impact of malicious nodes.

## 3. System and Threat Models

### 3.1. System Model

Suppose that there are several event areas of interest. To monitor relevant events in these areas, a cluster of static sensor nodes is deployed in each event area, a static BS is deployed to control the sensors and collect data from the sensors, and a group of forwarding nodes are deployed between each event area and the BS. The nodes and the BS can be deployed via aerial scattering or physical installation. Assume that each node can obtain its location information through the Global Positioning System (GPS) or a localization scheme [24,25], and all the nodes have the same sensing range *R_s_* and same communication range *R_c_*. Typically, *R_c_* is greater than 2*R_s_* [1]. Following deployment, the sensor nodes in each event area are organized into a cluster and each sensor node in the cluster is called a cluster node. Suppose that there are *Nc* cluster nodes in an event area, any of which can detect the events occurring in the event area and can participate in report generation. Besides, each cluster node can directly communicate with any other cluster node in the same event area. When an event occurs, *t* (*t* < *Nc*) cluster nodes in the event area are selected to jointly generate a signed event report, one of which is selected to be the CH. Finally, the CH transmits the signed event report to the BS through multi-hop routing.

**Figure 1:**
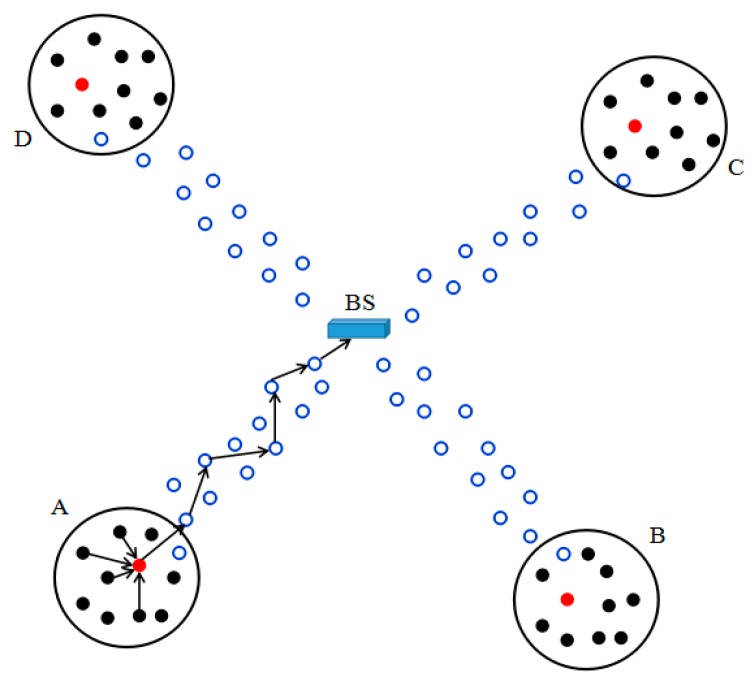
An example of a sensor network.

An example of a sensor network is shown in Figure 1. There are four event areas of interest to be monitored (the big black circles), denoted as A, B, C, and D respectively. A cluster of sensor nodes (the small circles filled with black color) are deployed in each event area. Suppose an event occurs in area A. The CH in area A (the small circle filled with red color) generates the event report, collects the partial signatures from *t* participating nodes in area A, and computes the final signature (called the cluster signature) for the report by using the *t* partial signatures. Then, the CH transmits the event report with its cluster signature to the BS through some forwarding nodes (the small blue circles).

### 3.2. Threat Models

Assume that sensor networks have a short safe bootstrapping phase right after network deployment, during which the adversary cannot compromise nodes or launch attacks. Subsequent to that, the adversary can compromise multiple nodes. However, the authors assume that the BS will not be compromised because it is usually well-secured. When a node is compromised, all the information it holds will also be compromised, and the adversary can take full control of it to launch different kinds of attacks. The following attacks are the focus of this paper:

- False report injection attack [5]. The adversary might control the compromised nodes to inject forged event reports, which might cause false alarms as well as the exhaustion of the constrained resources of the network.
- Report disruption attack [6]. The compromised sensor node might contribute invalid endorsement to the event report. As a result, the report of a real event will be dropped by some forwarding node or the BS. Additionally, the compromised sensor node might refuse to participate in report generation, thus the CH cannot collect enough legitimate endorsements for the event report. Furthermore, if a CH node is compromised, it might refuse to send out the signed event report or send out an event report with an illegitimate signature, resulting in the real event not being reported to the BS.
- Selective forwarding attack [6]. The compromised forwarding node might selectively drop the legitimate reports, which will severely damage data availability and disrupt the event report service.

The study objective is to design an effective scheme to mitigate the impact of these attacks. The authors assume that most *t* − 1 sensor nodes in an event area can be compromised in the study models, otherwise the adversary can successfully generate fake reports to fool the BS with non-existing events. Additionally, the authors assume that at least *t* sensor nodes in an event area are not compromised, otherwise the CH cannot generate legitimate reports for the real events. Therefore, at least 2*t* − 1 sensor nodes in an event area should be deployed—*Nc* ≥ 2*t* − 1.

## 4. The Proposed Scheme

### 4.1. Scheme Overview

A series of public key-based security mechanisms for WSNs is proposed in this paper, as well as finally developing a public key-based authentication and en-route filtering scheme, called PKAEF. This sub-section introduces the design ideas for these mechanisms in PKAEF.

#### 4.1.1. Design of Mechanism for Filtering False Report

The basic idea of the mechanism for filtering a false report is to generate a cluster signature for each event report which is cosigned by *t* cluster nodes in that event area so that any forwarding node with the cluster public key can verify the signed event report and drop the false one. 

To reduce the length of a signed report, the elliptic curve Pintsov–Vanstone signature scheme (ECPVSS) [21,22] is adopted, which is a message recovery signature scheme based on ECC (elliptic curve cryptography). Using ECPVSS, the signer will pick a random *k* and use *kP* for encrypting part of the report. A different random *k* is required for a different signature, otherwise, the adversary might easily infer the private key by capturing any two signatures generated from the same *k*. PDF [19] adopts the joint Shamir random secret sharing scheme [23] to share a different random *k* among the group of sensors each time, which will incur high communication overhead and time overhead. To address this problem, this study let each participating node directly pick a random *k_j_* and provide *k_j_P* to jointly construct *kP*. Compared to PDF, this method is simpler and incurs less communication consumption and time overhead.

Due to the threat of node compromise, any single node cannot be trusted to hold the private key used for signing the event reports. Therefore, this study adopts Shamir’s secret sharing technique [23] to share the private key. To confine the impact of a compromised node to its vicinity, the authors let each cluster share a unique private key (called a cluster private key) among the sensor nodes in that cluster (each cluster node holds a unique share of the cluster private key). Any *t* sensor nodes in a cluster can reconstruct the cluster private key collaboratively. Therefore, each event report will be cosigned by *t* cluster nodes in that event area. As long as less than *t* sensor nodes in a cluster are compromised, the adversary cannot obtain the cluster private key and cannot generate a valid cluster signature.

#### 4.1.2. Design of Mechanism for Resisting Report Disruption Attacks

The adversary might launch the report disruption attacks by controlling the compromised nodes to contribute invalid partial signatures. When a participating node provides an invalid partial signature, it will result in an invalid cluster signature being generated for the event report. Thus, the event report will be dropped by some forwarding node and unable to reach the BS. The existing PKC-based schemes do not provide any measure to verify the partial signatures. To defend against such attacks, the authors propose a verification mechanism to detect the invalid partial signatures, thereby avoiding generating an invalid cluster signature for the event report. 

The adversary might control a compromised node to refuse to participate in report generation, in an attempt to disturb the generation of a cluster signature. Once the CH detects that a participating node refuses to participate in report generation, or provides an invalid partial signature, it needs to request a trusted non-participating cluster node to join in report generation. Therefore, a substitution scheme is designed to ensure that enough cluster nodes are maintained to participate in report generation. Furthermore, if a CH node is compromised, it might refuse to send out the signed event report or send out an event report with an illegitimate signature, in an attempt to make the event report unable to reach the BS. The existing PKC-based schemes (DSEDA and PDF) do not provide any solution to such attacks. To resist such attacks, the authors propose an effective solution. Once the CH node has any malicious behavior, a new CH will substitute it and regenerate a signed event report.

#### 4.1.3. Design of Mechanism for Resisting Selective Forwarding Attacks

The multi-path routing protocol in [6] incurs high communication and computation overhead, while the report forwarding method in LEDS [11] incurs high computation overhead. To overcome these drawbacks, the authors have designed an effective report forwarding protocol that can defend against selective forwarding attacks with less communication and computation overhead. The main idea is that the sender first selects one upstream node to forward the report and, if the selected upstream node does not forward the report, the sender will inform another upstream node to verify and forward the report. Unless all the upstream nodes of the sender are compromised, the legitimate report ultimately will be forwarded to the BS. Compared with the methods in [6,11], the current scheme can reduce computation and communication overhead by reducing the number of nodes that verify and forward the report at each hop.

#### 4.1.4. Design of Mechanism for Malicious Nodes Detection and Isolation

To mitigate the impact of malicious nodes, the authors propose a trust value-based approach to identify and isolate the malicious nodes in the network. Each node establishes an empty isolation list and a trusted neighbor list to store the trust values of its neighbors, in which the trust values of all its neighbors are initialized to 1. The trust value of a node decides whether this node will be selected to participate in report generation or forward the reports. A node is considered to be trusted if its trust value is higher than the predefined threshold *T_T__v_*; otherwise, it is suspicious. When a node *v_i_* detects malicious behavior of its neighbor *v_k_*, it will decrease the trust value of *v_k_* (denoted as *TV_k_*) in its trusted neighbor list. When *v_i_* finds that *TV_k_* drops below *T_T__v_*, then *v_i_* will delete *v_k_* from the trusted neighbor list and add it into the isolation list to isolate it (*v_i_* will not transmit any message to *v_k_* or process any message from it). 

The reduction in trust values according to different malicious behaviors can be described as follows:

- During the report generation phase, if a participating node *v_j_* refuses to provide *k_j_P* or a partial signature, or provides an incorrect partial signature, then each cluster node will deduct 0.2 from *TV_j_*, and *v_j_* will be substituted.
- During the report generation phase, if the CH node *v_i_* refuses to broadcast *X*(*R*), or maliciously accuses any participating node of not providing *k_j_P* or a partial signature, or providing an incorrect partial signature, or *v_i_* refuses to send out the signed report, or sends out a report with an incorrect cluster signature, then each cluster node will deduct 0.2 from *TV_i_*, and a new CH node will be selected to regenerate a signed event report.
- If a forwarding node *v_t_* detects a false report sent by *v_k_*, then *v_t_* will deduct 0.1 from *TV_k_*.
- If a forwarding node *v_t_* maliciously refuses to forward the report from *v_k_*, then *v_k_* will deduct 0.1 from *TV_t_*.
- If a cluster node *v_k_* transmits a forged report with an incorrect cluster signature to the forwarding node *v_t_*, then *v_t_* and each cluster node will deduct 0.1 from *TV_k_*.

Note: (1) The first two malicious behaviors are more serious than the others, so a higher value should be deducted from the trust value of the malicious node correspondingly. (2) The reduction of trust value for a malicious behavior can be set according to the requirement of a specific application. As an example, if the network has low security requirements, a small value (0.02 for example) can be deducted from the trust value for a malicious behavior.

Next, the scheme will be introduced in detail.

### 4.2. Deployment and Initialization

A cluster of sensor nodes and a group of forwarding nodes are prepared for each event area to be monitored. The BS distributes a unique cluster ID for each cluster and generates a secret polynomial *f_Si_*(*x*) = *a*_0_ + *a*_1_*x* + *a*_2_*x*^2^ + … + *a_t_*_−1_*x^t^*^−1^ for each cluster, where *a*_0_, …, *a_t_*_−1_ are random numbers picked in the finite field F*p* (*p* is an odd prime number) and *S_i_* is the cluster private key which can be picked as *S_i_* = *a*_0_. Therefore, the corresponding cluster public key should be *Q_i_* = *S_i_P*, where *P* is the base point of order *q* in the group of points of the elliptic curve E(F*p*). Subsequent to that, the BS calculates the secret share of *S_i_* (denoted as *x_j_* = *f_si_*(*ID_j_*)) for each node *v_IDj_* in the cluster, then calculates *x_j_P* which will be used for verifying the partial signature of *v_IDj_*. Prior to network deployment, each cluster node *v_IDj_* has to be preloaded with its unique node ID (*ID_j_*), cluster ID, cluster public key *Q_i_*, its secret share *x_j_*, *x_k_P* of each other node *v_IDk_* in the same cluster, the elliptic curve domain parameters, and the system parameters (*t* and *T_T__v_*). The forwarding nodes will be preloaded with its unique node ID, cluster ID, *Q_i_*, the elliptic curve domain parameters, and the system parameters.

Note: (1) Each cluster node stores *x_k_P* of each other node *v_IDk_* in the same cluster so it can verify the partial signature of *v_IDk_*. (2) Having each cluster node store *x_k_P* rather than *x_k_* is to prevent the adversary from deriving *x_k_* from *x_k_P*. (3) Each cluster node holds a unique share of the cluster private key *S_i_*, and any *t* cluster nodes can jointly reconstruct *S_i_* by using a Lagrange interpolation $S_{i}=\sum_{j=1}^{t}l_{j}x_{j}$, where $l_{j}=\prod_{k=1,k\neq j}^{t}\frac{ID_{k}}{ID_{k}-ID_{j}}$ is the Lagrange coefficient. However, it is impossible to reconstruct *S_i_* with fewer than *t* cluster nodes. 

Following network deployment, each node calculates its *level* (its distance to the BS) as follows: the BS broadcasts a CL message including its level *Lev_BS* (*Lev_BS* = 0), asking each node to calculate its level. Once a node *v_i_* without an assigned level hears this message, it assigns its level *Lev_i* to be the level in the CL message plus one. Then, *v_i_* broadcasts the CL message with its own level. The CL message floods down until all nodes have been assigned a level. Subsequent to that, each node broadcasts its basic information {node ID, cluster ID, level, location} which will be stored by all of its neighbors. Then, each node transmits its location to the BS. Additionally, each node establishes an empty isolation list and a trusted neighbor list in which the trust values of all its neighbors are initialized to 1. Assume that the above procedure is safe during which no node is compromised. To further improve the security of the keys, the cluster public key and the share of cluster private key on each node can be updated periodically. One can refer to the literature [26,27] for the key management and distribution methods.

### 4.3. Report Generation

During this phase, the authors will discuss how the cluster nodes jointly generate a signed report for an event.

Suppose that there are *Nc* (*Nc* ≥ 2*t* − 1) cluster nodes in an event area, all of which can detect any event in that event area. When an event occurs, *t* of them should participate in report generation. Then, *t* out of *Nc* cluster nodes for generating the report should be chosen. Ferng et al. [1] designed a selection method, however, this method requires that the event values observed by different cluster nodes must be exactly the same but, in fact, the event values observed by different cluster nodes usually have some deviations. Thus, the *t* participating nodes selected by different cluster nodes might be different, which will affect the generation of the event report. To overcome this drawback, an improved method for selecting participating nodes is designed in the current study, as follows. 

When an event occurs, each cluster node in the event area sets a random timer. Upon the timer expiration, it will broadcast its observed values of the event in the form of {*E*, *L_e_*, *t_e_*}, where *E* is the event type, *L_e_* is the event location, and *t_e_* is the event time. When another cluster node finds that the broadcast values are consistent with what it observes within the predefined error range, it will record them and cancel its own timer. Subsequent to that, each cluster node sets a timer *TRG*, indicating that the report generation phase starts. The CH should send out the signed report before the timer expires. When a cluster node hears the correct signed report sent by the CH, it will cancel the timer *TRG*, indicating that the report generation phase ends. Additionally, each cluster node sets a variable *Fail_flag*, which is initialized to *false*, indicating that the report generation does not fail. Then, each cluster node detecting the same event uses a hash function to map {*E*, *L_e_*, *t_e_*} to a reference value *hv* within the same range of cluster node IDs. Then, the *t* trusted cluster nodes, whose IDs are closer to *hv* than the others, are picked to participate in report generation, among which the node whose ID is closest to *hv* becomes the CH. Each cluster node can determine the *t* IDs because each cluster node knows the range of cluster node IDs in the same event area. 

Following selection of the *t* participating nodes, each cluster node prepares an original report for the event in the form of *M* = {*CID*, *ID_ch_*, *E*, *L_e_*, *t_e_*}, where *CID* is the cluster ID, and *ID_ch_* is ID of the CH. Then, the event report *M* is divided into two parts: *C* and *V*. *C* holds {*ID_ch_*, *E*, *L_e_*}, while *V* holds {*CID*, *ID_ch_*, *L_e_*, *t_e_*}, for example. Since some redundant information is included in *C*, |*C*| + |*V*| ≥ |*M*|. Suppose the *t* participating nodes are *v_ID1_*, *v_ID2_*, …, *v_IDt_*, among which *v_IDi_* is the CH. Next, the *t* participating nodes perform the following steps to cosign the original event report: 

Step 1. The *t* participating nodes jointly construct *kP* for encrypting *C*. Each participating node *v_IDj_* (1 ≤ *j* ≤ *t*) picks a random *k_j_* in [1, *q* − 1] (*q* is the order of the elliptic curve) and sends *k_j_P* to the CH node *v_IDi_* rather than explicit *k_j_*, so that *v_IDi_* cannot derive *k_j_* from *k_j_P*. 

Step 2. When a participating node does not send *k_j_P* to *v_IDi_*, go to Step 5. Otherwise, *v_IDi_* computes *R* = *kP* = $\sum_{j=1}^{t}k_{j}P$ and broadcasts *X*(*R*) to the participating nodes, where *X*(*R*) is the value of the X-coordinate of *R*. Note that if *v_IDi_* refuses to broadcast *X*(*R*), it will not obtain partial signatures from the other participating nodes. Then, it might broadcast a substitution message, or refuse to send out the report, or send out a report with an incorrect signature. However, all these malicious behaviors will be detected by the other cluster nodes.

Step 3. Each participating node *v_IDj_* uses *X*(*R*) to generate *e = ENC*(*X*(*R*), *C*) and *d = H*(*e||V*), where *H* is a hash function (SHA–1 for example), || denotes concatenation, and the *ENC* denotes a symmetric-key encryption algorithm (AES, DEA, and RC5 for example). Subsequent to that, *v_IDj_* computes its share of the cluster signature (partial signature) *σ_j_ = x_j_l_j_d + k_j_* and sends it to *v_IDi_*. Note that during the report generation phase, each cluster node will listen to and temporarily store the information sent by the other cluster nodes, which will be used to identify malicious behaviors. 

Step 4. When *v_IDi_* detects a partial signature *σ_j_*, which is not from any of the *t* participating nodes, it will drop that *σ_j_* immediately. Otherwise, *v_IDi_* should verify *σ_j_* and drop the incorrect one in time. The authors have designed a verification scheme as follows:

① Compute *σ_j_P* (i.e., *σ_j_P* = (*x_j_l_j_d* + *k_j_*)*P* = *x_j_l_j_dP* + *k_j_P*);
② Compute *x_j_l_j_dP + k_j_P* by using *x_j_P* stored locally and *k_j_P* received from *v_IDj_* in Step 1;
③ Compare the results in ① and ②. When the result in ① is equal to that in ②, *σ_j_* is considered to be correct, otherwise *σ_j_* is considered to be incorrect.

When *v_IDi_* gets *t* correct partial signatures, go to Step 6. Otherwise, go to Step 5.

Step 5. Suppose that there are *z* (1 ≤ *z* < *t*) participating nodes to be substituted because they provided incorrect *σ_j_*, or they did not provide *k_j_P* or *σ_j_*. Then, what will substitute the *z* nodes to participate in report generation? The authors have designed a substitution scheme as follows.

First, *v_IDi_* broadcasts a substitution message SUBMSG, embedded with *ID_i_*, the reason code *rcd* (*rcd* is 0 if the *z* participating nodes will be substituted due to failure to provide *k_j_P*, or 1 if the *z* participating nodes will be substituted because they did not provide their partial signatures or provided incorrect partial signatures), *z*, and the IDs of the *z* participating nodes to be substituted. When a cluster node receives the SUBMSG message, it executes Algorithm 1 to process the message. Then, *v_IDi_* deducts 0.2 from the trust value of each participating node *v_IDm_* to be substituted. When *TV_IDm_* falls below *T_Tv_* and the number of ALARM messages for *v_IDm_* (denoted as *NA_IDm_*) is lower than *Nc*/2, *v_IDi_* will broadcast an ALARM message (embedded with *ID_i_* and *ID_m_*) for *v_IDm_* to inform other cluster nodes that *v_IDm_* is suspicious. *v_IDi_*, and each cluster node that hears the ALARM message, executes Algorithm 2 to process the ALARM message. When the number of trusted non-participating cluster nodes is less than *z*, which means there are not enough trusted participating nodes to cosign the report, then *v_IDi_* will set *Fail_flag* = *true*, cancel the timer *TRG*, and stop generating the signed report. Otherwise, the *z* trusted non-participating nodes whose IDs are closer to *hv* than the others will be selected to participate in report generation. When a new participating node does not send *k_j_P* to *v_IDi_*, *v_IDi_* performs the substitution scheme again. Otherwise, after receiving all the *k_j_P* from all the *z* new participating nodes, *v_IDi_* recomputes *R* = *kP* = $\sum_{j=1}^{t}k_{j}P$ and broadcasts X(*R*) to the current participating nodes. Then, each current participating node recomputes its partial signature *σ_j_* and sends it to *v_IDi_*. Following reception of a new partial signature from a current participating node, *v_IDi_* verifies it. When a current participating node does not provide its new partial signature or provides an incorrect partial signature, *v_IDi_* performs the substitution scheme again. Otherwise, go to Step 6. 

Step 6. *v_IDi_* sums the *t* correct partial signatures to produce the cluster signature: $\sigma =\sum_{j=1}^{t}\sigma_{j}$ mod *q*($\sigma =(\sum_{j=1}^{t}x_{j}l_{j}d+\sum_{j=1}^{t}k_{j})$ mod *q* = (*S_i_d* + *k*) mod *q*). Finally, *v_IDi_* sends the signed report {*V*, *e*, *σ*} to the BS through multi-hop routing. 

When a cluster node *v_k_* finds that the signed report sent by the CH is false (according to the steps S1–S4 in Section 4.4), or after the timer *TRG* ends, *v_k_* finds *Fail_flag* = *false* but does not hear the signed report sent by the CH, then *v_k_* performs Algorithm 3 to substitute the CH and to select a new CH to regenerate a signed report.


**Algorithm 1. Processing of SUBMSG Message**
/*Upon a cluster node *v_x_* receiving a SUBMSG message from the CH node v_IDi_*/
*flg* = *false*; // *flg* records whether the accusation in the SUBMSG message is true.
if (*rcd* = 0)
 if (the *z* nodes to be substituted did not send their *k_j_P* to *v_IDi_*) *flg* = *true*;
else if (*rcd* = 1)
 if (the *z* nodes to be substituted did not provide their *σ_j_* or provided incorrect *σ_j_*) *flg* = *true*;
if (*flg* = *false*)
 Perform Algorithm 3 to substitute *v_IDi_*;
else {
 for (each node *v_IDm_* to be substituted) {
  *TV_IDm_* = *TV_IDm_* − 0.2;
  if (*TV_IDm_* < *T_Tv_* && *NA_IDm_* < *Nc*/2) {
   Broadcast an ALARM message for *v_IDm_*;
   *v_x_*, and each cluster node that hears the ALARM message, executes Algorithm 2;
  }
 }//end for
 if (the number of trusted non-participating cluster nodes < *z*) {
  *Fail_flag* = *true*; // Report generation fails.
  Cancel the timer *TRG*;
 }
 else {
  Select the *z* trusted non-participating cluster nodes, whose IDs are closer to *hv* than the others, to participate in report generation;
  if (*v_x_* is a new participating node) {
   Pick *k_x_* in [1, *q* − 1] and send *k_x_P* to *v_IDi_*;
   Compute *σ_x_* after receiving *X*(*R*);
   Send *σ_x_* to *v_IDi_*;
  }
  else if (*v_x_* is a participating node that has not been substituted) {
   Recompute *σ_x_* after receiving *X*(*R*);
   Send *σ_x_* to *v_IDi_*;
  }
 }
}


**Algorithm 2. Processing of ALARM Message**
/*Upon a cluster node *v_x_* receiving an ALARM message for *v_y_* broadcast by node v_z_*/
if (it is the first ALARM message sent by *v_z_* for *v_y_*) *NA_y_* = *NA_y_* + 1;
if (*NA_y_* ≥ *Nc*/2) {
 Delete *v_y_* from the trusted neighbor list;
 Add *v_y_* into the isolation list;
}

### 4.4. Report Forwarding and En-Route Filtering

During this phase, two issues need to be addressed: (1) How to forward the signed report from the CH node to the BS? (2) How does a forwarding node verify the received report?

To resist selective forwarding attacks, the authors design a forwarding routing protocol. The sender, which could be a CH or a forwarding node, selects a trusted upstream node (*v_j_* for example) with the highest trust value, and transmits the signed event report to it. Each upstream node that hears the report will temporarily store the report. Then, the sender overhears the channel for a while. When the sender finds that *v_j_* does not forward the report, it will deduct 0.1 from *TV_j_* and inform another trusted upstream node whose trust value is higher than the others to verify and forward the report. When *TV_j_* falls below *T_Tv_*, the sender will delete *v_j_* from the trusted neighbor list and add it into the isolation list. Unless all the trusted upstream nodes of the sender are compromised, the legitimate report will ultimately be forwarded to the BS.


**Algorithm 3. Substitution of Cluster Head**
/*Substitution of the cluster head *v_IDi_* on a cluster node v_x_*/
*TV_IDi_* = *TV_IDi_* − 0.2;
if (*TV_IDi_* < *T_Tv_* && *NA_IDi_* < *Nc*/2) {
 Broadcast an ALARM message for *v_IDi_*;
 *v_x_*, and each cluster node that hears the ALARM message, executes Algorithm 2;
}
Reset the timer *TRG*;
Select the trusted non-participating cluster node, whose ID is closer to *hv* than the others (*v_t_* for example), to substitute *v_IDi_*;
Select the current participating node whose ID is closest to *hv* (*v_m_* for example) as the new CH;
if (*v_x_* is exactly *v_m_*) {
 if (*v_t_* does not provide *k_t_P*)
  Perform the proposed substitution scheme in Step 5;
 else {// *v_x_* has stored the *k_j_P* of the other participating nodes when they sent *k_j_P* to *v_IDi_*.
  Compute *R* and broadcast *X*(*R*);
  Receive and verify each new *σ_j_*;
  if (some current participating nodes do not provide their new *σ_j_* or provide incorrect *σ_j_*)
   Perform the proposed substitution scheme in Step 5;
 }
}
else if (*v_x_* is exactly *v_t_*) {
 Pick *k_x_* in [1, *q* − 1] and send *k_x_P* to *v_m_*;
 Compute *σ_x_* after receiving *X*(*R*) from *v_m_*;
 Send *σ_x_* to *v_m_*;
}
else if (*v_x_* is a participating node that has not been substituted) {
 Recompute *σ_x_* after receiving *X*(*R*) from *v_m_*;
 Send *σ_x_* to *v_m_*;
}

When a forwarding node *v_t_* receives a signed report {*V*, *e*, *σ*} from *v_k_*, where *V* = {*CID*, *ID_ch_*, *L_e_*, *t_e_*}, it will verify the report and drop the false one as follows:

S1. Check if *v_t_* has stored the pubic key *Q_CID_* of cluster *CID*; otherwise, drop the report and go to S5.
S2. Check the freshness of the report with the help of *t_e_* in the report; otherwise, drop the report and go to S5.
S3. When *v_t_* is a neighbor of the CH node *v_ID__ch_*, *v_t_* verifies the legitimacy of *v_ID__ch_*’s location *L_ID__ch_* stored locally by checking that d(*L_e_*, *L_ID__ch_*) ≤ *R_s_*. Otherwise, *v_t_* drops the report and goes to S5.
S4. Compute *d = H*(*e||V*), *R = σP* − *dQ_CID_*, and *C = ENC*^−1^(*X*(*R*), *e*), where *ENC*^−1^ denotes a decryption operation. Then, check that the {*ID_ch_*, *L_e_*} abstracted from *C* are the same as those in the report; otherwise, drop the report and go to S5.
S5. When the report is dropped, *v_t_* deducts 0.1 from *TV_k_*. When *TV_k_* falls below *T_Tv_*, *v_t_* deletes *v_k_* from the trusted neighbor list and adds it into the isolation list.
S6. When the report passes all the above checks, *v_t_* sends the report to the next hop by using the proposed forwarding routing protocol.

When a cluster node *v_a_* sends out a signed report, the selected forwarding node will verify the report and, at the same time, each cluster node *v_b_* that hears the report will also verify the report according to the steps S1–S4. When *v_b_* finds that the report is false, it will deduct 0.1 from *TV_a_*. When *TV_a_* falls below *T_Tv_* and *NA_a_* is lower than *Nc*/2, then *v_b_* will broadcast an ALARM message for *v_a_*. *v_b_*, and each cluster node that hears the ALARM message, executes Algorithm 2 to process the ALARM message. When *NA_a_* is greater than or equal to *Nc*/2, *v_b_* will delete *v_a_* from the trusted neighbor list and add it into the isolation list. 

### 4.5. Report Verification on the Base Station 

Upon receiving a signed report {*V*, *e*, *σ*}, where *V* = {*CID*, *ID_ch_*, *L_e_*, *t_e_*}, the BS verifies the signed report and recovers the original event report *M* from the legitimate signed report as follows:

- First, it checks the freshness of the report with the help of *t_e_*.
- Next, it checks that d (*L_e_*, *L_ID__ch_*) ≤ *R_s_*, where *L_ID__ch_* is the location of *v_ID__ch_* stored in the BS.
- It then verifies the cluster signature in the report using the pubic key *Q_CID_* of cluster *CID*. It first computes *d* = *H*(*e||V*), *R = σP* − *dQ_CID_*, and *C* = *ENC*^−1^(*X*(*R*), *e*), then checks that the {*ID_ch_*, *L_e_*} abstracted from *C* are the same as those in the report.

When any check is incorrect, the report will be dropped immediately. Otherwise, the BS recovers the original event report *M* = {*CID*, *ID_ch_*, *E*, *L_e_*, *t_e_*} from *C* and *V*.

## 5. Performance Analysis

The security performance, communication overhead, and storage overhead for DSEDA [1], PDF [19], and PKAEF are analyzed and compared in this section.

### 5.1. Security Analysis

The authors will compare the security performance of DSEDA, PDF, and PKAEF according to the following aspects: (1) security of private key; (2) resilience to false report injection attacks; (3) resilience to report disruption attacks launched by a non-CH node (a cluster node which is not a CH node); (4) resilience to report disruption attacks launched by a CH node; (5) resilience to selective forwarding attacks; (6) malicious nodes detection and isolation.

**Theorem** **1.** *PKAEF has a more secure private key than DSEDA and PDF, and PDF has a more secure private key than DSEDA.*

**Proof.**  Regarding DSEDA, each CH node holds a private key used for signing the event reports. The adversary just needs to compromise a CH to obtain its private key and it is easy for the adversary to find and capture a CH because the CHs and sensor nodes are heterogeneous in DSEDA. Concerning PDF, all nodes in the network share a unique system private key based on the Shamir’s secret sharing technique. Thus, the adversary needs to compromise *t* nodes to derive the system private key. Therefore, PDF has a more secure private key than DSEDA, however, the adversary just needs to compromise any *t* nodes anywhere in the network to derive the system private key in PDF. Looking at PKAEF, each cluster private key is shared by the sensor nodes in the cluster. Thus, to derive a cluster private key, the adversary has to compromise *t* sensor nodes in that cluster rather than in the whole network. Therefore, PKAEF has a more secure private key than PDF. Finally, PKAEF has a more secure private key than DSEDA and PDF, and PDF has a more secure private key than DSEDA. □

**Theorem** **2.** *PKAEF is more resilient to false report injection attacks than PDF and DSEDA, and PDF is more resilient to such attacks than DSEDA.*

**Proof.**  Regarding PDF, the adversary has to compromise at least *t* sensor nodes in the network to deduce the system private key; therefore, PDF can resist false report injection attacks when less than *t* sensor nodes in the network are compromised. Looking at PKAEF, the adversary has to compromise at least *t* sensor nodes in the same cluster to derive the cluster private key; therefore, PKAEF can resist false report injection attacks when less than *t* sensor nodes in the same cluster are compromised. It is generally more difficult for the adversary to capture *t* sensor nodes in the same cluster than to capture *t* sensor nodes in the whole network; therefore, PKAEF is more resilient to false report injection attacks than PDF. Concerning DSEDA, if a CH node is compromised it can successfully forge false reports with legitimate signatures using its private key. Since the CHs are fixed after deployment and the CHs and sensor nodes are heterogeneous, the adversary easily can find and capture the CHs; therefore, DSEDA is generally more vulnerable to false report injection attacks than PDF and PKAEF. To conclude, PKAEF is more resilient to false report injection attacks than PDF and DSEDA, and PDF is more resilient to such attacks than DSEDA. □

**Theorem** **3.** *PKAEF is more resilient to report disruption attacks launched by a non-CH node than DSEDA, and PDF cannot resist such attacks.*

**Proof.**  Regarding DSEDA, if a compromised non-CH node participating in report generation provides a secret share with an incorrect MAC, or refuses to provide a secret share, then an alternative secret share from a non-participating node will be sent to the CH. The CH cannot verify the correctness of a secret share, however. Thus, a compromised non-CH node participating in report generation might forge a wrong secret share and generate a correct MAC for it to pass the MAC verification of the CH. When the report with wrong secret shares reaches the BS, the BS will recover the wrong event information. To summarize, DSEDA only can combat report disruption attacks launched by a non-CH node to a certain extent. PKAEF provides a verification mechanism to detect the invalid partial signatures and a substitution scheme to maintain enough participating nodes for report generation. When a compromised non-CH node participating in report generation refuses to provide *k_j_P* or a partial signature, or provides an incorrect partial signature, then a non-participating cluster node will substitute it to participate in report generation. Therefore, PKAEF is more resilient to report disruption attacks launched by a non-CH node than DSEDA. PDF does not provide any measure to combat report disruption attacks launched by a non-CH node, so it cannot resist such attacks. □

**Theorem** **4.** *PKAEF can resist report disruption attacks launched by a CH node, while DSEDA and PDF cannot resist such attacks.*

**Proof.**  Looking at PKAEF, if a CH node refuses to send out the signed event report or sends out an event report with an illegitimate signature, then a new CH will substitute it, and a new signed event report will be generated. Therefore, PKAEF can resist report disruption attacks launched by a CH node. DSEDA and PDF, however, do not provide any measure to combat such attacks, so they cannot resist such attacks. □

**Theorem** **5.** *PKAEF can resist selective forwarding attacks, while DSEDA and PDF cannot resist such attacks.*

**Proof.**  PKAEF provides an efficient report-forwarding protocol. When the selected forwarding node does not forward the report, the sender will try other trusted upstream nodes until the report is forwarded; therefore, PKAEF can resist selective forwarding attacks. DSEDA and PDF, however, do not provide any mechanism to combat selective forwarding attacks, so they cannot resist such attacks. □

**Theorem** **6.** *PKAEF can detect and isolate the malicious nodes while DSEDA and PDF cannot.*

**Proof.**  PKAEF provides a trust value-based approach to detect and isolate the malicious nodes. Looking at PKAEF, each node establishes a trusted neighbor list to store the trust values of its neighbors. When a node has any malicious behavior, its trust value will be reduced, and it will be isolated if its trust value falls below the predefined threshold. DSEDA and PDF, however, do not provide any mechanism to detect and isolate the malicious nodes. □

Finally, the security performance of DSEDA, PDF, and PKAEF is summarized in Table 1, in which “Poor” means the scheme cannot resist such attacks, while “High”, “Moderate” and “Low” indicate the relative ability of these schemes to resist such attacks.

### 5.2. Overhead Analysis

#### 5.2.1. Communication Overhead

1. Communication overhead for report generation

The communication overhead for report generation is measured as the message overhead during the report generation phase. A 160-bit elliptic curve is chosen for DSEDA, PDF, and PKAEF. Then, the lengths of the base point, public key, private key, and the order are 40 bytes, 40 bytes, 20 bytes, and 20 bytes respectively. 

During the report generation phase in DSEDA, each of the *T* participating sensor nodes needs to send its {*E_u_*, *u*, MAC_*Ku,CH*_(*E_u_*, *u*)} to the CH. Suppose that the lengths of node ID, *E_u_*, and MAC are 1 byte, 8 bytes, and 8 bytes, respectively, and (*T*, *t*) = (6, 5). Then, the total message overhead for report generation in DSEDA is 102 bytes.

Regarding PDF, the message overhead for report generation includes four parts: (a) to share a random *k*, each of the *t* participating nodes needs to send a share of secret *f_si_*(*s_j_*) to each of the other *t* − 1 participating nodes. The message overhead is *t* × (*t* − 1) × 20 bytes. (b) The *t* − 1 participating nodes except the CH, need to send *k_i_l_i_P* to the CH. The message overhead is (*t* − 1) × 40 bytes. (c) The CH needs to broadcast *R*. The message overhead is 40 bytes. (d) The *t* − 1 participating nodes, except the CH, need to send their partial signatures to the CH. The message overhead is (*t* − 1) × 20 bytes. When *t* = 5, as set in DSEDA, then the total message overhead for report generation in PDF is 680 bytes. When PDF uses pre-computation to reduce the communication overhead for *k* sharing, then the message overhead in part (a) can be saved, and the total message overhead for report generation will be 280 bytes.

Regarding PKAEF, the message overhead for report generation includes four parts: (a) a cluster node broadcasts its observed event values {*E*, *L_e_*, *t_e_*} for selecting participating nodes. (b) The *t* − 1 participating nodes except the CH, need to send *k_j_P* to the CH. The message overhead is (*t* − 1) × 40 bytes. (c) The CH needs to broadcast *X*(*R*). The message overhead is 20 bytes. (d) The *t* − 1 participating nodes, except the CH, need to send their partial signatures to the CH. The message overhead is (*t* − 1) × 20 bytes. When the lengths of *E*, *L_e_*, and *t_e_* are 4 bits, 20 bits, and 4 bytes respectively, and *t* = 5 as set in DSEDA, then the total message overhead for report generation in PKAEF is 267 bytes.

It can be seen that the message overhead for report generation in PDF is the highest, most of which is incurred by the sharing of *k*. PKAEF reduces the message overhead for report generation by making each participating node pick a random *k_j_* rather than *k* sharing and, by making the CH broadcast *X*(*R*) rather than *R*. The message overhead for report generation in DSEDA is the lowest, since the event report in DSEDA is signed by the CH rather than the *t* participating nodes, however, that the private key is kept by a single node will result in poor security of the private key. 

Note, when the adversary launches the report disruption attacks, DSEDA will incur an additional message overhead for compensating the lack of secret shares, and PKAEF will incur an additional message overhead for performing the substitution scheme or selecting a new CH. However, the additional communication overhead in DSEDA or PKAEF is acceptable, since it is associated with great advantages in data availability and security strength.

2. Communication overhead for forwarding a report

The communication overhead for forwarding a report is affected by the length of the signed report message. The longer the length of the signed report message, the higher the communication overhead for forwarding a report at each hop. 

Suppose the format of the original event report in PDF is the same as that in PKAEF, *M* = {*CID*, *ID_ch_*, *E*, *L_e_*, *t_e_*}, and each original event report *M* is divided into *C* = {*ID_ch_*, *E*, *L_e_*} and *V* = {*CID*, *ID_ch_*, *L_e_*, *t_e_*}. Suppose that the lengths of *CID*, *ID_ch_*, *E*, *L_e_*, and *t_e_* are 4 bits, 8 bits, 4 bits, 20 bits, and 32 bits respectively, then the size of *M* is 9 bytes. The 32-bit RC5 algorithm is adopted as the symmetric-key encryption algorithm in DSEDA, PDF, and PKAEF. Then, the length of a signed report message in PDF and PKAEF is 39 bytes (including an extra 3-byte header). *CID* is not necessary in DSEDA, thus the format of the event report in DSEDA could be *S* = {*ID_ch_*, *E*, *L_e_*, *t_e_*}, and the size of *S* is about 8 bytes. DSEDA utilizes the bloom filter to reduce the size of IDs of the *T* participating nodes to 3 bytes and adopts the partial message recovery algorithm to reduce the signature overhead to 26 bytes. Suppose that the length of secret share *S_u_* is 8 bytes and (*T*, *t*) = (6, 5). Then, the length of a signed report message in DSEDA is 80 bytes (including an extra 3-byte header). Letting (*T*, *t*) = (5, 4), then the length of a signed report message in DSEDA is 72 bytes (including an extra 3-byte header).

It can be seen that the length of a signed report message in DSEDA is longer than that in PDF or PKAEF, which will result in higher communication overhead for forwarding a report at each hop.

#### 5.2.2. Storage Overhead

Compare the storage overhead of DSEDA, PDF, and PKAEF. Here, the storage overhead of the elliptic curve domain parameters and the system parameters is overlooked, which are almost the same for all three algorithms. Thus, the key storage overhead is the main consideration. Assume that DSEDA, PDF, and PKAEF are all applied in the sensor networks shown in Figure 1. Suppose that there are *Nc* sensor nodes in a cluster, and a 160-bit elliptic curve is chosen for DSEDA, PDF, and PKAEF. 

Regarding DSEDA, each sensor node stores two unique symmetric keys shared with the sink and one symmetric key shared with the CH. Each CH stores two symmetric keys shared with the sink, *Nc* − 1 symmetric keys shared with the sensor nodes in the cluster, and a public/secret key pair. Suppose that the length of a symmetric key is 8 bytes, then the average key storage overhead of a cluster node in DSEDA is *ST_DSEDA* = (3 × (*Nc* − 1) × 8 + (*Nc* − 1 + 2) × 8 + 40 + 20)/*Nc* bytes = 32 + 44/*Nc* bytes.

Regarding PDF, each cluster node stores the system public key (40 bytes) and its secret share of the system private key (20 bytes). Furthermore, when PDF uses pre-computation to reduce the communication overhead for *k* sharing, then each cluster node is preloaded with the shares of different *k* during the deployment. Suppose that each cluster node stores the shares of different *k* for generating *m* reports, then the corresponding storage overhead is *m* × *Nc* × 20 bytes. Thus, the total storage overhead of a cluster node in PDF is *ST_PDF* = 60 + *m* × *Nc* × 20 bytes. When PDF does not use the pre-computation method, then *ST_PDF* = 60 bytes.

Regarding PKAEF, each cluster node stores the cluster public key (40 bytes), its secret share of cluster private key (20 bytes), and *x_k_P* of the other *Nc* − 1 cluster nodes ((*Nc* − 1) × 40 bytes). In total, the key storage overhead of a cluster node in PKAEF is *ST_PKAEF* = 40 + 20 + (*Nc* − 1) × 40 bytes = 20 + *Nc* × 40 bytes.

Generally, DSEDA has a higher storage efficiency than PDF and PKAEF, but this is at the expense of the security of the private key. When PDF uses the pre-computation method, then PKAEF is more storage-efficient than PDF. Otherwise, PDF is more storage-efficient than PKAEF, but at the expense of higher communication overhead for report generation. Looking at PKAEF, storing *x_k_P* of the other nodes in the cluster will increase the storage overhead, but this is desirable since it supports PKAEF to verify partial signatures.

Finally, the comparison results of overhead are provided in Table 2, in which more “*” indicates higher overhead, and the key storage overhead of PDF is “**” when PDF does not use the pre-computation method, or “****” when PDF uses the pre-computation method.

## 6. Performance Evaluation

The authors evaluate the performance of PDF [19], DSEDA [1], and PKAEF through simulation experiments based on the WSN simulator in the literature [12], which was developed in C++ language on a PC with dual-core 1.6 GHz CPU and 8 GB memory. The authors simulated a network with two event areas, and randomly deployed 10 sensor nodes in each event area (*Nc* = 10). According to the distance from the center of the event area to the BS, two different scenarios were simulated. In Scenario 1, the distance from the center of the event area to the BS was 300 m, and 20 forwarding nodes were deployed between the BS and each event area. In Scenario 2, the distance from the center of the event area to the BS was 600 m, and 40 forwarding nodes were deployed between the BS and each event area. The other simulation parameters are provided in Table 3. 

In the simulation, the SECG recommended 160-bit elliptic curve (secp160r1) was selected and the 32-bit RC5 algorithm was used as the symmetric-key encryption algorithm. The evaluation focuses on the following aspects: energy expenditure for report generation; performance of false report filtering; resilience to report disruption attacks; resilience to selective forwarding attacks. All the experiment results were averaged over 1000 runs. 

### 6.1. Energy Expenditure for Report Generation

The authors compare the performance of PDF, DSEDA, and PKAEF in terms of energy expenditure for report generation. Since how to select the *t* participating nodes is not mentioned in PDF, the authors adopted the same selection method used in PKAEF for PDF in the simulation and overlooked the energy expenditure incurred by selecting participating nodes for PDF. Moreover, PDF provides two methods of *k* sharing: one is to precompute and prestore the shares of *k* (PDF is denoted as PDF_NSk in this case), and the other is to distribute the shares of *k* for each event report (PDF is denoted as PDF_Sk in this case). 

**Figure 2:**
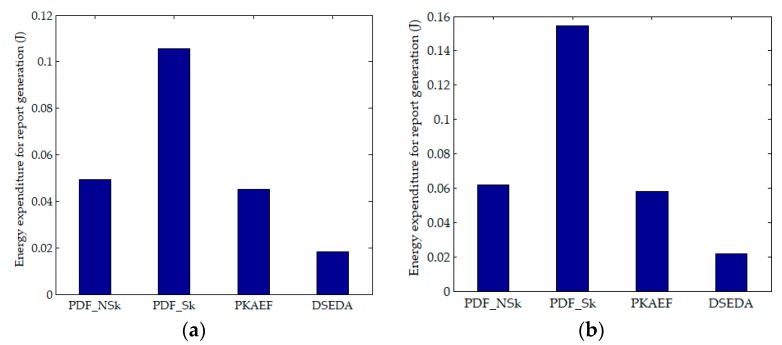
Energy expenditure for report generation. (**a**) in Scenario 1; (**b**) in Scenario 2.

Figure 2a,b shows the results of energy expenditure for generating a signed report in Scenario 1 and Scenario 2, respectively. It can be seen that the energy expenditure of PDF_Sk is the highest because it requires more energy to distribute the shares of *k*. DSEDA has the lowest energy expenditure for report generation. This is because the participating nodes in DSEDA do not sign the report jointly and the energy expenditure for sending the endorsement information from the participating nodes to the CH is low. PKAEF is more energy efficient than PDF_NSk, which benefits from broadcasting *X*(*R*) rather than *R*. These results are consistent with the previous analysis.

### 6.2. Performance of False Report Filtering

The authors compare the performance of PDF, DSEDA, and PKAEF on the aspect of false report filtering. During each experiment, *cn* cluster nodes in the two event areas were compromised randomly and each of them injected a forged report into the network. Regarding DSEDA, if the CH was compromised, it was assumed that the other compromised cluster nodes also could obtain the private key of the CH, and then they could forge reports with correct signatures. Concerning PDF, a forwarding node verifies the signature of a report with the probability of *p_f_*. The authors simulated *p_f_* = 100% and *p_f_* = 50% for PDF, and PDF is denoted as PDF_100 and PDF_50, respectively. The lengths of a signed report message in PDF and PKAEF were 39 bytes in both Scenario 1 and Scenario 2, while the lengths of a signed report message in DSEDA were 72 bytes in Scenario 1 and 80 bytes in Scenario 2. The authors evaluate the performance of false report filtering based on two metrics: the en-route filtering probability (the percentage of dropped false reports by the forwarding nodes), and the filtering energy expenditure (the average energy expenditure for filtering out a false report since it is injected into the network until it is dropped).

**Figure 3:**
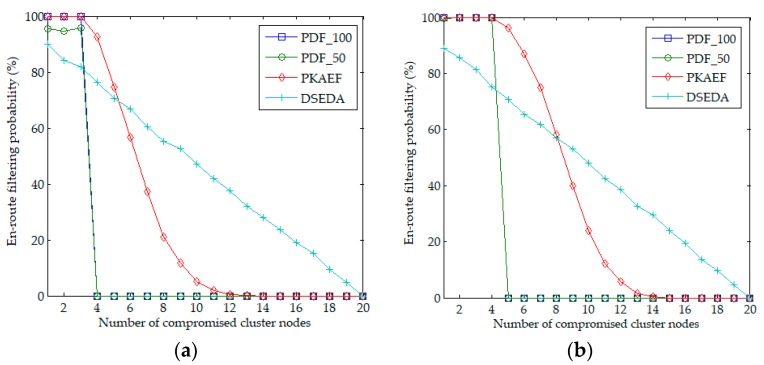
En-route filtering probability as a function of the number of compromised cluster nodes. (**a**) in Scenario 1; (**b**) in Scenario 2.

Figure 3a,b shows the results of the en-route filtering probability when *cn* varied from 1 to 20 in Scenario 1 and Scenario 2, respectively. The authors observe that when *cn* < *t*, the en-route filtering probabilities of PDF_100 and PKAEF are 100%, which are higher than those of PDF_50 and DSEDA. This is because when *cn* < *t*, the adversary cannot infer the private key in PDF_100, PDF_50, and PKAEF. The forged reports in PDF_100 and PKAEF will be filtered out at their 1-hop forwarding nodes, while the forged reports in PDF_50 can escape the verification of each forwarding node with a probability of 50%, some of which will reach the BS. Regarding DSEDA, a compromised node might be a CH, then the adversary can obtain its private key and the forged reports in that cluster will pass the verification of forwarding nodes and reach the BS. Therefore, the en-route filtering probability of DSEDA is lowest when *cn* < *t*. When *cn* ≥ *t*, the en-route filtering probabilities of PDF_100 and PDF_50 are 0%, because the adversary can infer the system private key in PDF_100 and PDF_50, and thus can forge reports with correct signatures, all of which will reach the BS. The en-route filtering probabilities of DSEDA and PKAEF gradually decrease to 0% with the increase of *cn*. When *cn* < 6 in Scenario 1 (*cn* < 9 in Scenario 2), PKAEF has a higher en-route filtering probability than DSEDA, otherwise DSEDA has higher en-route filtering probability. However, it should be noted that a random node capture was adopted in the experiments, which is very beneficial for DSEDA. Since the CHs in DSEDA are fixed, the adversary would prefer to capture the CHs rather than the non-CH nodes to obtain the private keys, in fact.

**Figure 4:**
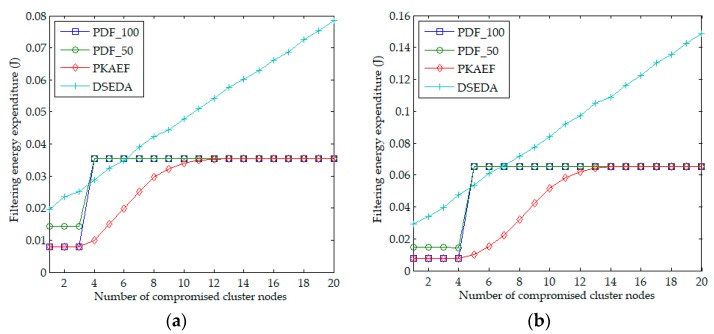
Filtering energy expenditure as a function of the number of compromised cluster nodes. (**a**) in Scenario 1; (**b**) in Scenario 2.

Figure 4a,b shows the results of the filtering energy expenditure when *cn* varied from 1 to 20 in Scenario 1 and Scenario 2, respectively. It is observed that when *cn* < *t*, PDF_100 and PKAEF have the same filtering energy expenditures and both outperform PDF_50 and DSEDA because the en-route filtering probabilities of PDF_100 and PKAEF are highest and the forged reports in PDF_100 and PKAEF are filtered out within one hop. When *cn* ≥ *t*, PDF_50 and PDF_100 have the same filtering energy expenditures, which are much higher than those when *cn* < *t*. This is because the adversary can infer the system private key in PDF_50 and PDF_100, and the forged reports with correct signatures will reach the BS. Regarding PKAEF, the adversary has to randomly compromise more cluster nodes to infer a cluster private key; therefore, when *cn* ≥ *t*, PKAEF has lower filtering energy expenditure than PDF_50 and PDF_100 before the adversary infers the cluster private key. Furthermore, it can be seen that the filtering energy expenditure of DSEDA is highest in most cases, even though the en-route filtering probability of DSEDA is higher than those of PDF_50, PDF_100 and PKAEF. This is because the length of a signed report message in DSEDA is much longer than that in PDF_50, PDF_100, and PKAEF.

### 6.3. Resilience to Report Disruption Attacks

The authors compare the performance of PDF, DSEDA, and PKAEF in terms of resilience to report disruption attacks. During each experiment, *cn* cluster nodes in the two event areas were compromised randomly. When a compromised cluster node was selected as a participating node, it would launch a report disruption attack; otherwise it would not launch a report disruption attack. Concerning PDF and PKAEF, a compromised non-CH node participating in report generation could refuse to provide *k_j_P* or a partial signature or provide an incorrect partial signature. Considering DSEDA, a compromised non-CH node participating in report generation could contribute a secret share with an incorrect MAC or refuse to participate in report generation (DSEDA is denoted as DSEDA_MAC in this case), or it could forge a false secret share *S_u_* and generate a correct MAC for it (DSEDA is denoted as DSEDA_Su in this case). When a CH node was compromised in PDF, DSEDA, and PKAEF, it could refuse to send out the signed report or send out a report with an incorrect signature. The authors evaluate the resilience to report disruption attacks according to the proportion of correct signed reports sent out by the CH.

**Figure 5:**
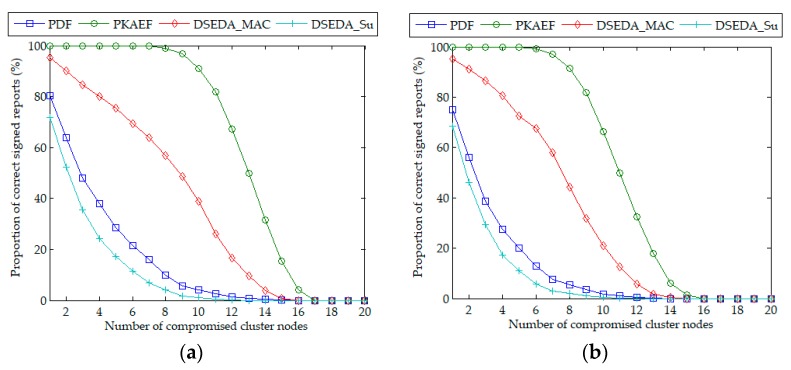
Proportion of correct reports as a function of the number of compromised cluster nodes. (**a**) in Scenario 1; (**b**) in Scenario 2.

Figure 5a,b shows the results of the proportion of correct signed reports when *cn* varied from 1 to 20 in Scenario 1 and Scenario 2, respectively. It can be observed that, when arranged according to the proportion of correct signed reports, the sequence is PKAEF, DSEDA_MAC, PDF, and DSEDA_Su from the highest to the lowest in order. PKAEF has the highest proportion of correct signed reports, which benefits from the verification of partial signatures, the detection of malicious behavior, and the substitution mechanism. DSEDA_Su has the lowest proportion of correct signed reports because it does not provide any method to detect false *S_u_*. Furthermore, in both scenarios, all the proportions of correct signed reports gradually decrease to 0% with the increase of *cn*. This is because, in DSEDA, if the number of compromised cluster nodes in an event area is greater than *Nc*−*T*, then there are not enough normal cluster nodes to generate correct signed reports; while in PDF and PKAEF, if the number of compromised cluster nodes in an event area is greater than *Nc*−*t*, then there are not enough normal cluster nodes to generate correct signed reports.

### 6.4. Resilience to Selective Forwarding Attacks

The authors compare the performance of PDF, DSEDA, and PKAEF in terms of resilience to selective forwarding attacks. Additionally, to compare the proposed report forwarding protocol and multi-path routing protocol, the authors also simulated PKAEF adopting a 2-path routing protocol, denoted as PKAEF_2MP. During each experiment, *fn* forwarding nodes around an event area were compromised randomly. When a forwarding node was compromised, it would drop each report that passed by it, with the probability of *ps*. The authors evaluate the resilience to selective forwarding attacks based on data availability, which is measured as the proportion of available reports (the proportion of normal reports that can reach the BS). The proportion of available reports is affected by *fn* and *ps*. The larger the *fn* is, the lower the proportion of available reports is. The larger the *ps* is, the lower the proportion of available reports is.

**Figure 6:**
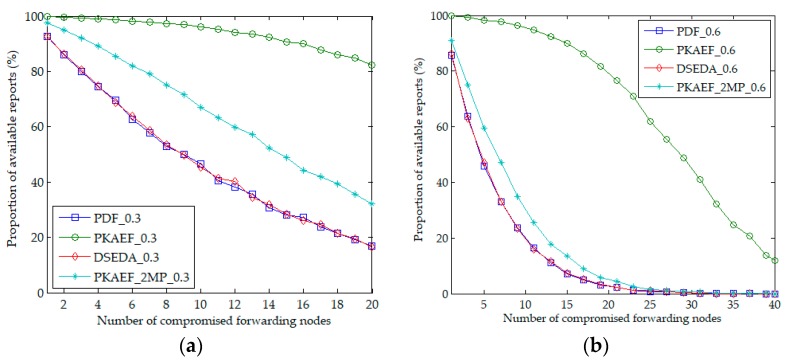
Proportion of available reports as a function of the number of compromised forwarding nodes. (**a**) in Scenario 1; (**b**) in Scenario 2.

Figure 6a,b shows the proportions of available reports affected by the number of compromised forwarding nodes in Scenario 1 and Scenario 2, respectively. During the experiments, *fn* varied from 1 to 20 in Scenario 1, and from 1 to 40 in Scenario 2. The probability *ps* was set to 0.3 and 0.6 in Scenario 1 and Scenario 2, respectively. Shown in Figure 6, the proportions of available reports of PDF, DSEDA, PKAEF_2MP, and PKAEF decrease as *fn* increases. The proportions of available reports of PDF and DSEDA are almost the same, both lower than those of PKAEF_2MP and PKAEF, as they do not provide any strategy to resist selective forwarding attacks. The proportion of available reports of PKAEF_2MP is higher than those of PDF and DSEDA, but lower than that of PKAEF.

**Figure 7:**
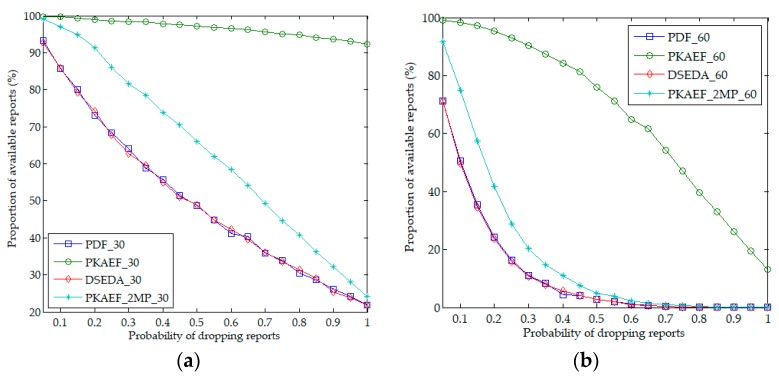
Proportion of available reports as a function of the probability of dropping reports. (**a**) in Scenario 1; (**b**) in Scenario 2.

Figure 7a,b shows the results of the proportion of available reports when *ps* varied from 0.05 to 1.0 in Scenario 1 and Scenario 2, respectively. During the experiments, the number of compromised forwarding nodes was set to 30% and 60% of the total number of forwarding nodes in Scenario 1 and Scenario 2, respectively—*fn* = 6 in Scenario 1 and *fn* = 24 in Scenario 2. It can be observed that the proportions of available reports of PDF, DSEDA, PKAEF_2MP, and PKAEF decrease as *ps* increases. The proportions of available reports of PDF and DSEDA are almost the same, both lower than those of PKAEF_2MP and PKAEF, and PKAEF has the highest proportion of available reports. Therefore, PKAEF is more resilient to selective forwarding attacks than PDF and DSEDA, and this study’s report forwarding protocol is superior to the 2-path routing protocol.

Considering the report forwarding protocol of PKAEF, once the sender finds that the selected forwarding node does not forward the report, it will select another trusted upstream node with the highest trust value to forward the report, rather than randomly selecting an upstream node to forward the report. To compare the performance of the two methods: randomly selecting forwarding nodes and selecting forwarding nodes based on trust values, the authors performed simulation experiments for PKAEF by using these two methods respectively. PKAEF is denoted as PKAEF_TV when the forwarding nodes are selected based on trust values and denote PKAEF as PKAEF_NTV when the forwarding nodes are randomly selected. Three groups of experiments were carried out based on the value of *ps*, where the values of *ps* were set to 0.2, 0.6, and 1.0 respectively. Each group of experiments was run 1000 times and, in each run, 50 reports in an event area were sent to the BS by different cluster heads. The authors evaluate the performance of PKAEF_TV and PKAEF_NTV according to the average energy expenditure of forwarding a report at each hop.

**Figure 8:**
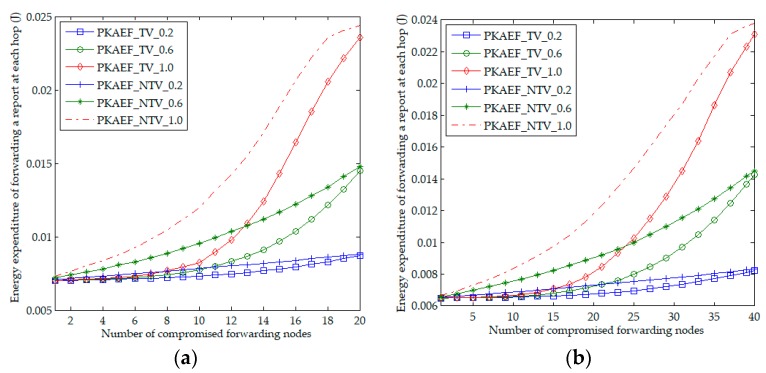
Energy expenditure for forwarding a report at each hop as a function of the number of compromised forwarding nodes. (**a**) in Scenario 1; (**b**) in Scenario 2.

Figure 8a,b shows the energy expenditures of forwarding a report at each hop affected by *fn* and *ps* in Scenario 1 and Scenario 2, respectively. It can be observed that, for both PKAEF_NTV and PKAEF_TV, the energy expenditure of forwarding a report at each hop increases with the increase of *fn* and *ps*. This is because when *fn* and *ps* are larger, the sender needs to choose other upstream nodes to forward the report more times, resulting in higher energy expenditure. Happening in both scenarios, PKAEF_NTV requires a higher energy expenditure of forwarding a report at each hop than PKAEF_TV, regardless of whether *ps* is 0.2, 0.6, or 1.0. This is because, in PKAEF_TV, the sender prefers forwarding nodes with high trust values which are less likely to be compromised nodes, and thus the report can be successfully forwarded earlier. However, in PKAEF_NTV, the randomly selected forwarding nodes are more likely to be compromise nodes, and the sender needs to try more forwarding nodes for successful forwarding, resulting in higher energy consumption. It can be seen that, compared with the method of randomly selecting the forwarding nodes, the method of selecting the forwarding nodes based on trust values is a better choice for PKAEF.

## 7. Conclusions

A public key-based authentication and en-route filtering scheme (PKAEF) is proposed in this paper. PKAEF integrates a mechanism for verifying the partial signatures and a substitution mechanism to substitute the malicious cluster nodes or CHs; thus, PKAEF can resist report disruption attacks. Additionally, PKAEF contains an effective report-forwarding protocol and an en-route filtering scheme, which makes it able to combat selective forwarding attacks and false data injection attacks. Moreover, PKAEF provides a trust value-based mechanism for detecting and punishing the malicious nodes, which can mitigate the impact of malicious nodes. Both theoretical analysis and simulation results demonstrate that, in most cases, PKAEF outperforms DSEDA and PDF in terms of security, filtering efficiency, and data availability.

## Figures and Tables

**Table 1:** Comparison of security performance.

Security Performance	PKAEF	PDF	DSEDA
Security of private key	High	Moderate	Low
Resilience to false report injection attacks	High	Moderate	Low
Resilience to report disruption attacks launched by a non-cluster head (CH) node	High	Poor	Low
Resilience to report disruption attacks launched by a CH node	High	Poor	Poor
Resilience to selective forwarding attacks	High	Poor	Poor
Malicious nodes detection and isolation	Yes	No	No

*PKAEF (public key-based authentication and en-route filtering scheme); PDF (public-key based false data filtering scheme); DSEDA (digital signature assisted end-to-end data authentication scheme).*

**Table 2:** Comparison of overhead.

Overhead	PKAEF	PDF	DSEDA
Communication overhead for report generation	**	***	*
Communication overhead for forwarding a report	*	*	**
Key storage overhead	***	**/****	*

*PKAEF (public key-based authentication and en-route filtering scheme); PDF (public-key based false data filtering scheme); DSEDA (digital signature assisted end-to-end data authentication scheme).*

**Table 3:** Simulation parameters.

Parameter	In Scenario 1	In Scenario 2
Communication range *R_c_*	80 m	80 m
Sensing range *R_s_*	40 m	40 m
*T* in DSEDA	5	6
*t*	4	5
Power consumption of transmitting 1 byte	16.25 uJ	16.25 uJ
Power consumption of receiving 1 byte	12.5 uJ	12.5 uJ
